# New soft X-ray beamline BL07LSU at SPring-8

**DOI:** 10.1107/S1600577513034796

**Published:** 2014-02-08

**Authors:** Susumu Yamamoto, Yasunori Senba, Takashi Tanaka, Haruhiko Ohashi, Toko Hirono, Hiroaki Kimura, Masami Fujisawa, Jun Miyawaki, Ayumi Harasawa, Takamitsu Seike, Sunao Takahashi, Nobuteru Nariyama, Tomohiro Matsushita, Masao Takeuchi, Toru Ohata, Yukito Furukawa, Kunikazu Takeshita, Shunji Goto, Yoshihisa Harada, Shik Shin, Hideo Kitamura, Akito Kakizaki, Masaharu Oshima, Iwao Matsuda

**Affiliations:** a Synchrotron Radiation Laboratory, Laser and Synchrotron Research Center, The Institute for Solid State Physics, The University of Tokyo, 5-1-5 Kashiwanoha, Kashiwa 277-8581, Japan; b Synchrotron Radiation Research Organization, The University of Tokyo, 7-3-1 Hongo, Bunkyo-ku, Tokyo 113-8656, Japan; c Japan Synchrotron Radiation Research Institute, SPring-8, Sayo, Hyogo 679-5198, Japan; d RIKEN SPring-8 Center, Koto 1-1-1, Sayo, Hyogo 679-5148, Japan; e Department of Applied Chemistry, School of Engineering, The University of Tokyo, 7-3-1 Hongo, Bunkyo-ku, Tokyo 113-8656, Japan

**Keywords:** soft X-ray beamline, figure-8 undulator, segmented cross undulator, polarization control, high energy resolution, high brilliance, plane-grating monochromator, materials science

## Abstract

A new soft X-ray beamline, BL07LSU, has been constructed at SPring-8 to perform advanced soft X-ray spectroscopy for materials science. The beamline is designed to achieve high energy resolution (*E*/Δ*E*> 10000) and high photon flux [>10^12^ photons s^−1^ (0.01% bandwidth)^−1^] in the photon energy range 250–2000 eV with controllable polarization. To realise this state-of-the-art performance, a novel segmented cross undulator was developed and adopted as a light source. The details of the undulator light source and beamline monochromator design are described. The achieved performance of the beamline, such as the photon flux, energy resolution and the state of polarization, is reported.

## Abstract 2

The design and performance of a new soft X-ray beamline BL07LSU at SPring-8 are described. The combination of a novel segmented cross undulator and beamline monochromator has achieved high energy resolution (*E*/Δ*E* > 10000) and high photon flux [>10^12^ photons s^−1^ (0.01% bandwidth)^−1^] in the photon energy range 250–2000 eV with fully controllable polarization.

## 1. Introduction   

Materials science is one of the fastest growing areas of research today. The increasing importance of materials science mirrors society’s strong demand towards developing novel materials that will solve energy and environmental problems. To accomplish this goal, it is necessary to have a fundamental understanding of how the functions of materials are related to their atomic, molecular and electronic structures.

Synchrotron-based soft X-ray spectroscopy has proven to be a powerful experimental technique for studying the atomic, molecular and electronic structures of materials (Nilsson *et al.*, 2008 ▶). The energy range of soft X-rays covers the *K*-edge absorption of the most abundant elements on Earth (*e.g.* C, N, O, Si) and the *L*-edge absorption of the industrially important transition metals (Ti, V, Cr, Mn, Fe, Co, Ni, Cu and Zn). Soft X-ray spectroscopy has many unique characteristics, such as element specificity, chemical specificity and surface sensitivity, which make them versatile for application in a wide range of scientific fields including surface chemistry, environmental science and magnetism. Owing to its importance, soft X-ray spectroscopy beamlines have been constructed at synchrotron radiation (SR) facilities worldwide (Amemiya *et al.*, 1996 ▶, 2002 ▶; Amemiya & Ohta, 2004 ▶; Mase *et al.*, 2010 ▶; Toyoshima *et al.*, 2011 ▶, 2013 ▶; Saitoh *et al.*, 2000 ▶; Ohashi *et al.*, 2007 ▶; Oura *et al.*, 2007 ▶; Takahashi *et al.*, 2005 ▶; Chen & Sette, 1989 ▶; Quaresima *et al.*, 1995 ▶; Cvetko *et al.*, 1997 ▶; Bluhm *et al.*, 2006 ▶; Beutier *et al.*, 2007 ▶; Polack *et al.*, 2010 ▶; Strocov *et al.*, 2010 ▶, 2011 ▶).

Three keywords in today’s science and technology are ‘smaller’, ‘faster’ and ‘more complex’. In our everyday technologies such as computers and mobile phones, we have witnessed the components of these devices becoming increasingly smaller. Furthermore, nanotechnology allows the measurement and manipulation of materials on the atomic and molecular scale. Concurrent with the decrease in the spatial scale, a faster time scale is required in both science and technology. The intrinsic time scale for dynamical processes in nanotechnology goes down to the time scale of atoms, electrons and spins, which is typically in the femtosecond (fs; 10^−15^ s) range; the vibration of atoms in a molecular chemical bond is 10–100 fs. The last keyword, ‘more complex’, is required because complexity lies, for example, in non-equilibrium states such as photo-excited states in photocatalysts, as well as in heterogeneous catalysts. Heterogeneous catalysts are complex materials. Real industrial catalysts often consist of very small crystallites in the nanometer range (nanoparticles). In nanoparticle catalysts, the active sites of catalytic reactions are specific sites on the surface at an atomic scale, such as defect sites (*e.g.* steps, kinks). Therefore, chemists would ideally be able to perform single-site and single-molecule spectromicroscopy to monitor chemical transformations at a specific active site. In addition, the complexity of heterogeneous catalysts is increased by the fact that the surface structure and surface chemical states of catalysts are readily changed by their reactive environment (*e.g.* gas pressure and temperature), and thus have to be studied directly under complex environments (Weckhuysen, 2003 ▶; Bañares, 2005 ▶). As seen above, the three keywords are closely connected with each other.

To perform advanced soft X-ray spectroscopy for materials science, a new soft X-ray beamline, BL07LSU, has been constructed at SPring-8 by the University of Tokyo in collaboration with SPring-8. This soft X-ray beamline is designed to achieve high energy resolution (*E*/Δ*E* > 10000) and high photon flux [>10^12^ photons s^−1^ (0.01% bandwidth)^−1^] in the photon energy range 250–2000 eV with controllable polarization (Senba *et al.*, 2011 ▶). To realise the state-of-the-art performance described above, a novel segmented cross undulator was developed and adopted as a light source. Three permanent endstations were developed to conduct advanced soft X-ray spectroscopy corresponding to the three keywords ‘smaller’, ‘faster’ and ‘more complex’.

This paper is structured as follows. The design and performance of the novel undulator light source are described in §2, including the details of the polarization analysis. In §3, the design and performance of the beamline monochromator (photon flux and energy resolution) are presented. In §4, the characteristics of the endstations at the beamline are briefly described. A summary of the paper is given in §5.

## 2. Undulator light source   

### 2.1. Background   

Before introducing the undulator light source for SPring-8 BL07LSU, it is worthwhile to give a brief overview of undulator light sources capable of polarization control and/or switching. One of the most popular undulator light sources capable of polarization control in the soft X-ray region is the APPLE-II undulator or an elliptically polarized undulator (EPU) (Sasaki *et al.*, 1993 ▶; Sasaki, 1994 ▶). Polarization control in this undulator is achieved by mechanically moving its permanent magnet arrays. Because of the limitation on the speed of the mechanical movement, the frequency of polarization switching is typically less than 0.1 Hz. A scheme for achieving faster polarization switching is to use twin undulators with kicker magnets (Hara *et al.*, 1998 ▶, 2003 ▶; Muro *et al.*, 2005 ▶; Sawhney *et al.*, 1997 ▶; Amemiya *et al.*, 2013 ▶); two undulators with different polarizations are placed in tandem, and one of them is selected to emit SR by modulating an electron orbit using kicker magnets. The frequency of polarization switching is typically less than ∼10 Hz. The disadvantage of the twin undulator scheme is that the light source point is shifted by the polarization switching. For long undulators, the displacement of the light source point also becomes large. It has been pointed out that the displacement of the source point during polarization switching may lead to different intensities and photon energies between switched polarizations (Amemiya *et al.*, 2013 ▶). In addition, the electron orbit in the storage ring could be greatly distorted by kicker magnets. Another scheme to realise fast polarization switching is the cross undulator (CU) proposed by Kim (1984 ▶). In the original CU scheme, two planar undulators are placed in tandem with a crossed configuration. A phase shifter (PS) installed between the two undulators is used to introduce an optical phase delay between the horizontal and vertical electric fields by creating a bump orbit in an electron path. This allows for the generation of various polarization states such as circular polarizations and tilted linear polarizations. The polarization switching frequency in the CU can reach 1 kHz when electromagnets are used as a PS (Kim, 1984 ▶). However, the unfortunate drawback of the CU is that the achievable degree of circular polarization is limited by the angular spread of the electron beam and the acceptance of the beamline.

### 2.2. Design   

**Figure 1:**
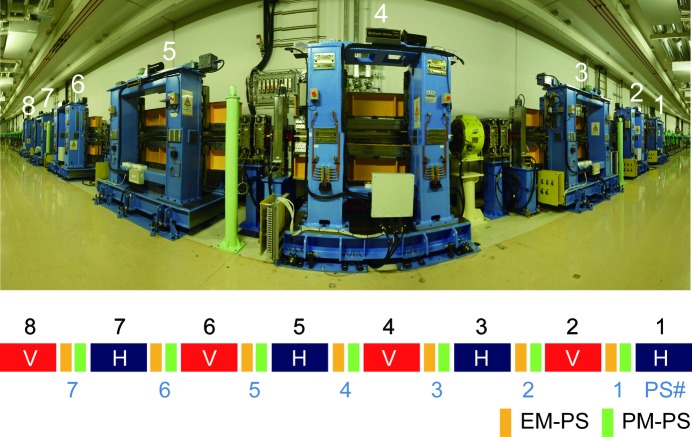
Overview photograph and schematic of the segmented cross undulator installed at a long straight section of SPring-8 BL07LSU. It consists of eight segments of figure-8 undulator and seven phase shifters (PSs). Four undulator segments generate horizontally polarized radiation at the fundamental radiation (shown in the schematic as H), and the other four segments generate vertically polarized radiation (shown in the schematic as V). Two types of PS are installed: permanent-magnet phase shifter (PM-PS) and electromagnet phase shifter (EM-PS). Note that the ID segments and PSs in the segmented cross undulator are in a straight line although the photograph shows visual distortion to keep a wide panoramic view of the 27 m-long undulator.

To overcome the challenges described above (*i.e.* to achieve a high degree of polarization with the capability of polarization control and fast polarization switching), a new insertion device (ID) has been designed and developed for SPring-8 BL07LSU. This novel ID is based on a segmentation concept proposed at SPring-8 (Tanaka & Kitamura, 2002 ▶, 2004 ▶) and is referred to as a segmented cross undulator (SCU). The SCU for SPring-8 BL07LSU consists of eight ID segments and seven PSs, and its total length is 27 m. Fig. 1 ▶ shows a photograph and a schematic of this SCU. Four ID segments generate horizontally linearly polarized radiation at the fundamental radiation (horizontal ID segment), and the other four ID segments generate vertically linearly polarized radiation (vertical ID segment). The horizontal and vertical ID segments are placed alternately. Circularly polarized radiation can be obtained by superposing horizontally and vertically linearly polarized radiation, and the helicity of the circularly polarized radiation can be changed by the PSs. The PSs are functionally equivalent to single-period undulators and are inserted between the ID segments to adjust the relative phase of the SR emitted from each segment by changing the path length of the electron orbit between ID segments with a local orbit bump. The PS has two important roles: (i) to maximize the photon flux by phase matching ID segments with the same polarization, and (ii) to control the polarization states by phase shifting between ID segments with different polarizations. Two types of PSs are installed: permanent-magnet phase shifters (PM-PSs) and electromagnet phase shifters (EM-PSs). To change the magnetic field of the PSs, the magnet gap is varied in the PM-PSs, while the coil current is altered in the EM-PSs. By using the EM-PSs, fast switching of polarization states becomes possible.

**Figure 2:**
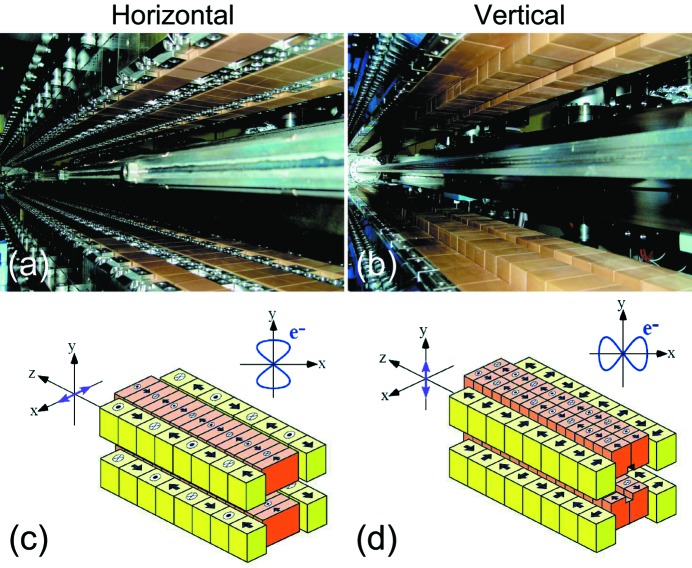
Photographs and schematic illustrations of horizontal (*a* and *c*) and vertical (*b* and *d*) figure-8 undulators at SPring-8 BL07LSU. In the schematics of the magnetic structures, the direction of the magnetic fields is shown on each magnet. Note that the period of the side-row magnets is twice that of the center-row magnets. In the horizontal figure-8 undulator, the electron trajectory resembles the figure of eight (8); in the vertical figure-8 undulator, it resembles the figure of infinity (∞).

Next, we take a more detailed look at each ID segment in the SCU for SPring-8 BL07LSU. A figure-8 undulator that was proposed and developed at SPring-8 is employed in each ID segment (Tanaka & Kitamura, 1995 ▶, 1996*a* ▶,*b* ▶; Tanaka *et al.*, 1998 ▶, 1999 ▶). As mentioned earlier, two types of undulator which generate opposite polarization states are required in the SCU concept; in the SCU for SPring-8 BL07LSU they are horizontal figure-8 and vertical figure-8 undulators. Photographs and schematic illustrations of these two types of figure-8 undulators are shown in Fig. 2 ▶. The electron trajectory in the horizontal figure-8 undulator literally resembles the figure of eight (8), whereas the electron trajectory in the vertical figure-8 undulator resembles the figure of infinity (∞). For the electron trajectory in the horizontal figure-8 undulator, the period in the *y* direction is twice that in the *x* direction, which is traced back to the magnet arrangement in Fig. 2(*c*) ▶, where the period of the side-row magnets, which are responsible for the horizontal magnetic field, is twice that of the center-row magnets creating the vertical magnet field. The main parameters of the horizontal and vertical figure-8 undulators are summarized in Table 1 ▶. Both have the same magnetic period of 100 mm but different numbers of periods *N* (*N* = 26 for horizontal and *N* = 20 for vertical ID segments), resulting in different total lengths of 2.6 and 2.0 m for the horizontal and vertical figure-8 undulators, respectively. The minimum gaps in the horizontal and vertical figure-8 undulators for generating the fundamental photon energy of 250 eV are 28 and 20 mm, respectively. The total lengths of the horizontal and vertical ID segments differ for the following reason. In the design stage of the SCU for BL07LSU, it was realised that the injection efficiency may become worse when all the ID segments are enabled because of coupling between the vertical deflection induced by the horizontal ID field and the large vertical betatron function in the long straight section where the SCU is located. Therefore, it was decided to shorten the vertical ID segment to secure extra space to install special octupole magnets (Soutome & Takao, 2013 ▶) to correct for this effect at the expense of the photon flux of the vertical polarization component. The smaller period number of vertical ID segments does not decrease the degree of circular polarization greatly (less than 0.03); the degree of circular polarization at this beamline is kept higher than 0.90 as will be shown in §2.3.2.

The important characteristic of the figure-8 undulator is the low power density at the on-axis undulator radiation cone (Tanaka & Kitamura, 1995 ▶, 1996*a* ▶,*b* ▶; Tanaka *et al.*, 1998 ▶, 1999 ▶). The heat load on the beamline optics from unnecessary high harmonics becomes a big problem for a soft X-ray beamline at a high-energy storage ring, which is exactly the case for our beamline at the 8 GeV storage ring at SPring-8. The use of an APPLE-II/EPU undulator (Sasaki *et al.*, 1993 ▶; Sasaki, 1994 ▶) is not feasible because of the heat load on the beamline optics. The other unique feature of the figure-8 undulator is that it has half-integer harmonics (*n* = 1/2, 3/2, 5/2,…) in addition to the integer harmonics (*n* = 1, 2, 3,…); the polarization of the half-integer harmonics is orthogonal to that of the integer harmonics (Tanaka & Kitamura, 1995 ▶, 1996*a* ▶,*b* ▶; Tanaka *et al.*, 1998 ▶, 1999 ▶). Thus, the polarization of the half-integer harmonics becomes vertical if the polarization of the integer harmonics is horizontal.

A variety of polarization states of SR can be generated by the SCU for SPring-8 BL07LSU: linear (horizontal, vertical and variable angles of the polarization plane) polarizations and circular (left, right and elliptical) polarizations. When a single type of ID segment (horizontal or vertical) in the SCU is enabled, the available SR polarization is either horizontal or vertical linear polarization. When both types of ID segment in the SCU are enabled, various polarizations are available as a function of the relative phase shift between the horizontal and vertical electric field vectors (Born & Wolf, 1999 ▶; Hecht, 2002 ▶). The relative phase shift can be controlled by the PSs. Consequently, the SCU for SPring-8 BL07LSU can be operated in three different operation modes: (i) horizontal/vertical linear polarization mode, (ii) various polarization mode, and (iii) fast polarization switching mode. For (i), the linear polarization mode, four ID segments having either horizontal or vertical polarization are employed with PM-PSs. Either horizontal or vertical linearly polarized SR is available. For (ii), the various polarization mode, all eight ID segments are used with PM-PSs. Circular polarization, elliptical polarization and tilted linear polarizations with variable angles of the polarization plane become available. Linear polarizations with variable angles of the polarization plane might be useful for accurately determining the orientation of molecular adsorbates on surfaces using polarization-dependent X-ray absorption spectra (Stöhr, 1992 ▶). In (iii), the fast polarization switching mode, all eight ID segments are used with EM-PSs. Switching between the polarizations available in the various polarization mode (ii) becomes possible; for example, switching between left- and right-handed circular polarizations and between tilted linear polarizations can be performed. The fast polarization switching mode aims for switching at a frequency of >10 Hz. EM-PS operation at 50 Hz succeeded in our preparatory experiment outside of a storage ring. The advantage of the SCU for SPring-8 BL07LSU in polarization switching is that the source point is not displaced during polarization switching in the various polarization mode. Note that when four ID segments are used in the horizontal/vertical linear polarization mode (i), the source point of the ID is shifted by 3.4 m along the beam axis.

### 2.3. Performance   

In this subsection, we describe the performance of the undulator light source. In the SCU, the PS has two important roles: (i) to maximize the photon flux by phase matching of the ID segments with the same polarization, and (ii) to control the polarization states by phase shifting between ID segments with different polarizations. In the following, the performance of the PS (photon flux and polarization) is addressed.

#### 2.3.1. Photon flux   

**Figure 3:**
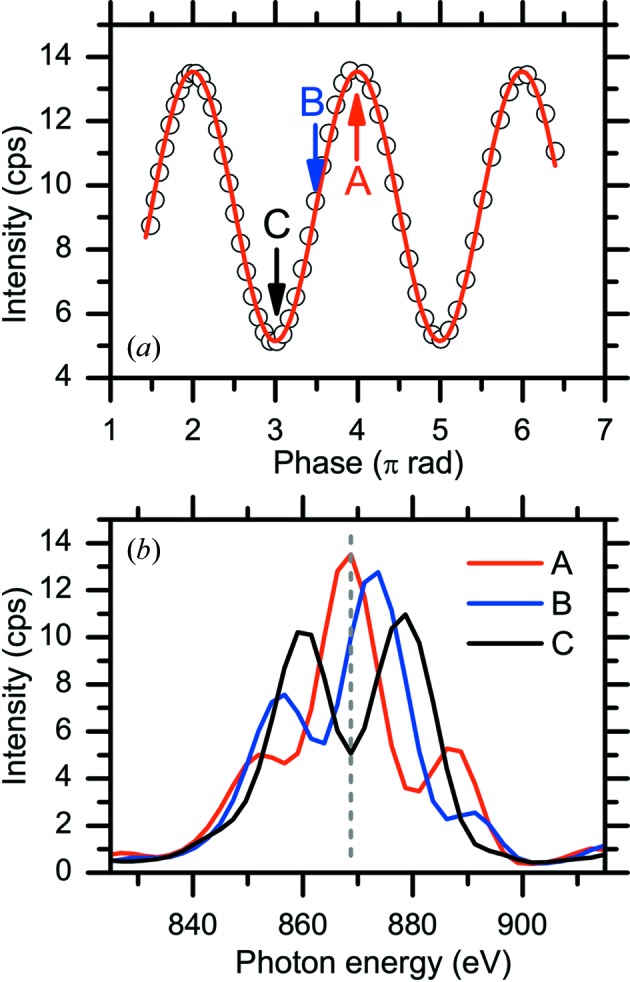
(*a*) Photon flux intensity at 870 eV from two horizontal figure-8 undulators as a function of phase shifter gap. The horizontal axis is converted from the phase shifter gap to the relative phase shift in units of π rad. (*b*) ID spectra at the representative relative phase shift points indicated in (*a*).

**Figure 4:**
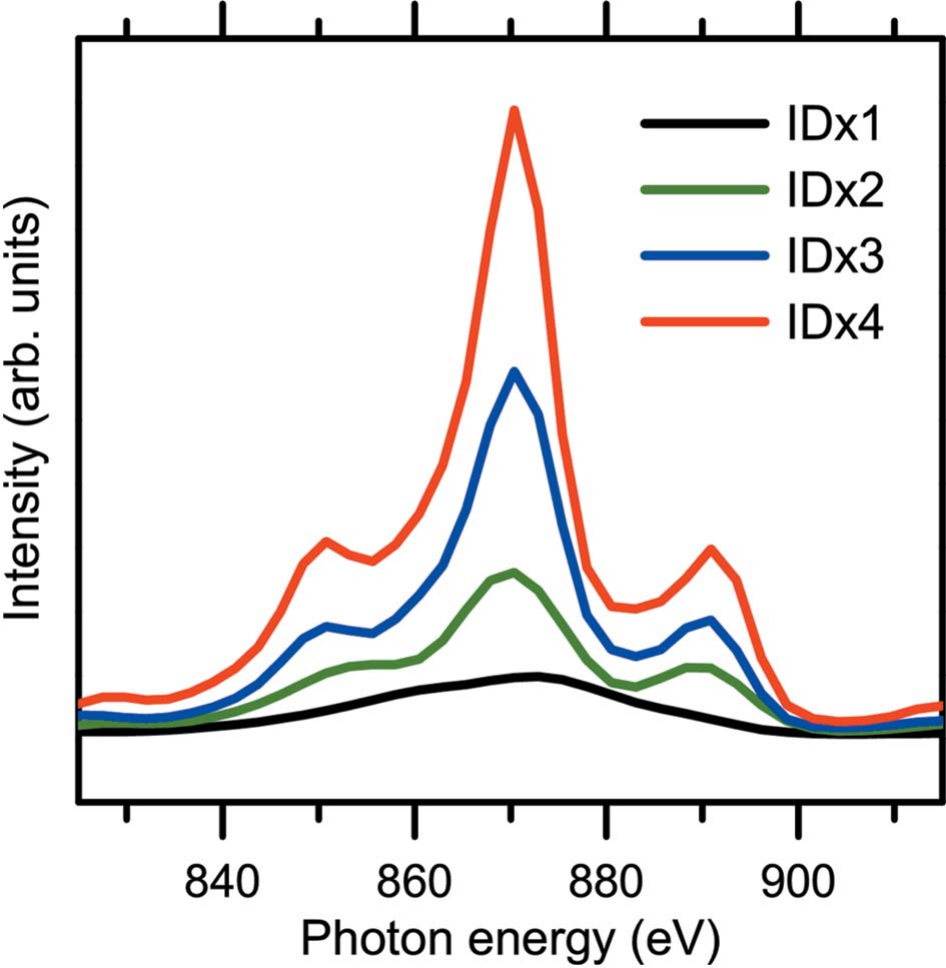
ID spectra as a function of ID segment number at 870 eV: ID × 1 (H1), ID × 2 (H1 + H3), ID × 3 (H1 + H3 + H5) and ID × 4 (H1 + H3 + H5 + H7). ID spectra were measured using a 600 lines mm^−1^ grating with front-end slit opening of 0.5 mm × 0.5 mm and exit slit width of 100 µm.

Fig. 3 ▶ demonstrates the operation of the PS to maximize the photon flux. As shown in Fig. 3(*a*) ▶, the photon flux intensity from two horizontal ID segments shows a sinusoidal variation as a function of PM-PS gap. The ID spectrum when the two ID segments are in phase exhibits higher photon flux at the central energy with a narrower spectrum width, as seen in Fig. 3(*b*) ▶. Fig. 4 ▶ shows ID spectra of the horizontal figure-8 undulator segments as a function of ID segment number. The peak width of the ID spectra becomes narrower as the number of ID segments increases. The intensity of the ID spectra at the central energy (*h*ν = 870 eV) increases as follows: ID × 1 (H1) 1.00 (the intensity of SR from one segment is normalized to unity), ID × 2 (H1 + H3) 2.71, ID × 3 (H1 + H3 + H5) 6.00 and ID × 4 (H1 + H3 + H5 + H7) 10.27. The photon flux density increases in proportion to the square of the number of periods of the ID segments, *N* ^2^ (Attwood, 1999 ▶). The experimentally observed decrease of the proportional factor from *N* ^2^ originates from the emittance of the electron beam and the angular acceptance of the beamline. The photon flux intensity of each ID segment is affected by the angular acceptance of the beamline that is determined by the distance from each ID segment to a front-end slit and the opening size of the front-end slit. The photon flux intensity from each horizontal ID segment with front-end slit opening of 0.5 mm × 0.5 mm at *h*ν = 870 eV is as follows (ID spectra are not shown in the paper): ID × 1 (H1) 1.00, ID × 1 (H3) 1.10, ID × 1 (H5) 1.80, ID × 1 (H7) 2.06. As the ID segments approach the front-end slit (*i.e.* moving downstream from H1 to H7), the transmission of SR through the front-end slit increases. Photon flux intensities of various polarizations and their comparison with the simulated values will be discussed in §2.3.2(ii) and in Table 2.

#### 2.3.2. Polarization   

Polarization is one of the most important characteristics of SR. The unique SCU at SPring-8 BL07LSU allows full control of the SR polarization, as discussed in §2.2. It is therefore important to characterize the state of polarization by a polarization analysis. In this section, we describe the polarimeters that were developed to conduct the polarization analysis and present the results of polarization analysis of SR light at SPring-8 BL07LSU.

**Figure 5:**
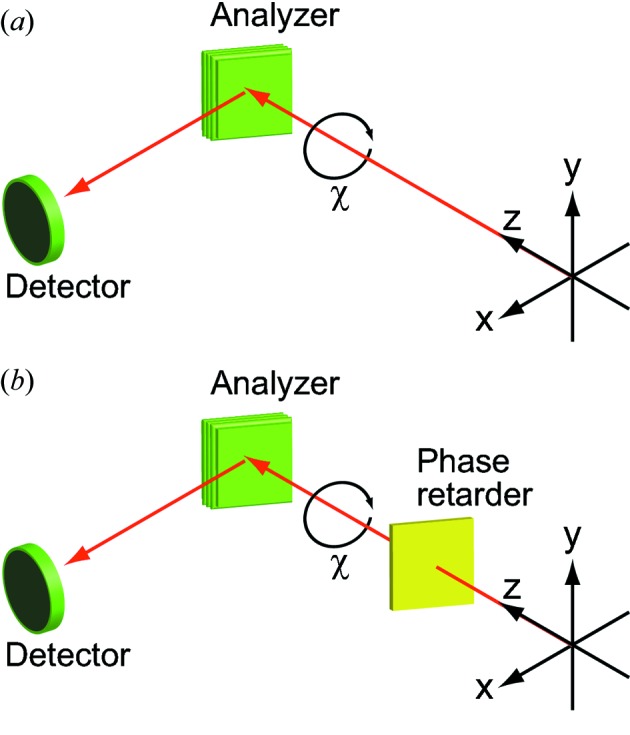
Two optical configurations for polarization measurement at SPring-8 BL07LSU.

(i) *Polarimeter*. Fig. 5 ▶ shows the two optical configurations of the polarimeter used to evaluate the polarization state of SR light. The optical configuration in Fig. 5(*a*) ▶ is used to measure the degree of linear polarization and consists of an analyzer and a detector. The degree of circular polarization can be evaluated by adding a phase retarder, which changes circularly polarized light into linearly polarized light, as shown in Fig. 5(*b*) ▶. The polarimeter is based on a rotating analyzer method (Kimura *et al.*, 1993 ▶; Schäfers *et al.*, 1999 ▶). As shown in Fig. 5(*a*) ▶, the incident SR beam is reflected by the analyzer to the detector. A multilayer mirror is typically used as the analyzer to increase the reflectivity. The reflectivity of the multilayer analyzer is measured as a function of azimuth angle χ by rotating the analyzer.

The polarimeter with the optical configuration in Fig. 5(*a*) ▶ has been developed at SPring-8 BL07LSU. The multilayer analyzers are made of W/B_4_C with *N* = 100 periods for periodic distances of *d* = 2.99 nm (for *h*ν = 300 eV) or 1.19 nm (for *h*ν = 720 eV) (NTT-AT Corporation). The detector is a microchannel plate (MCP) (Hamamatsu Photonics Corporation, MCP F4655). This home-built polarimeter is so compact that all the mechanical parts are mounted on an ICF253 flange, and the polarimeter can be permanently installed at the beamline. In addition, it is equipped with a load lock chamber, which allows the storage of five multilayer mirrors and their exchange without breaking vacuum.

The polarimeter with the optical configuration in Fig. 5(*b*) ▶ was developed by some of our co-authors to conduct full polarization analysis (Kimura *et al.*, 1995 ▶, 2004 ▶, 2005 ▶; Hirono *et al.*, 2004 ▶, 2005 ▶). The phase retarder of Sc/Cr multilayers (*N* = 300 and *d* = 3.15 nm) in a transmission configuration acts as a quarter-wave plate that changes circularly polarized light into linearly polarized light. The transformed linearly polarized light is then characterized by the Sc/Cr multilayer analyzer (*N* = 200 and *d* = 2.24 nm) and the MCP detector. Note that the optical configuration in Fig. 5(*a*) ▶ can also be adopted by removing the phase retarder from the beam path. Details of the polarimeter used for full polarization analysis are available in previous publications (Kimura *et al.*, 2004 ▶, 2005 ▶; Hirono *et al.*, 2004 ▶, 2005 ▶). Note that all the polarization analyses presented here were conducted at a sample position after the beamline optics (*i.e.* the mirrors and grating).

The polarization state of the incident light, *S*, is expressed by the normalized Stokes vector as
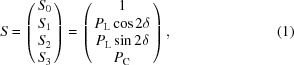
where *P*_L_ is the degree of linear polarization, *P*_C_ is the degree of circular polarization, and δ is the azimuth angle of the major axis of the polarization ellipse of the incident light. *P*_L_ and δ can be determined by the rotation analyzer method as follows. The intensity of the reflected light in the rotation analyzer method, *I*(χ), follows the modified Malus’s law expressed as follows (Kimura *et al.*, 1993 ▶; Hecht, 2002 ▶; Imazono *et al.*, 2009 ▶),
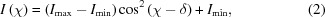
where *I*_max_ and *I*_min_ are the maximum and minimum values of the reflected light, respectively. χ is the azimuth angle of the analyzer. The contrast factor *C* is defined as
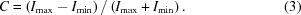
The polarizing ability of the analyzer is called the polarizance *Z* and is defined as
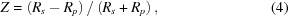
where *R*_*p*_ and *R*_*s*_ are the reflectivity of *p*- and *s*-polarized light, respectively. If the value of *Z* is known, the degree of linear polarization *P*_L_ can be derived by dividing the contrast factor *C* by *Z*,
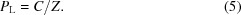
For the perfect analyzer (*Z* = 1), the degree of linear polarization *P*_L_ equals the contrast factor *C*.

**Figure 6:**
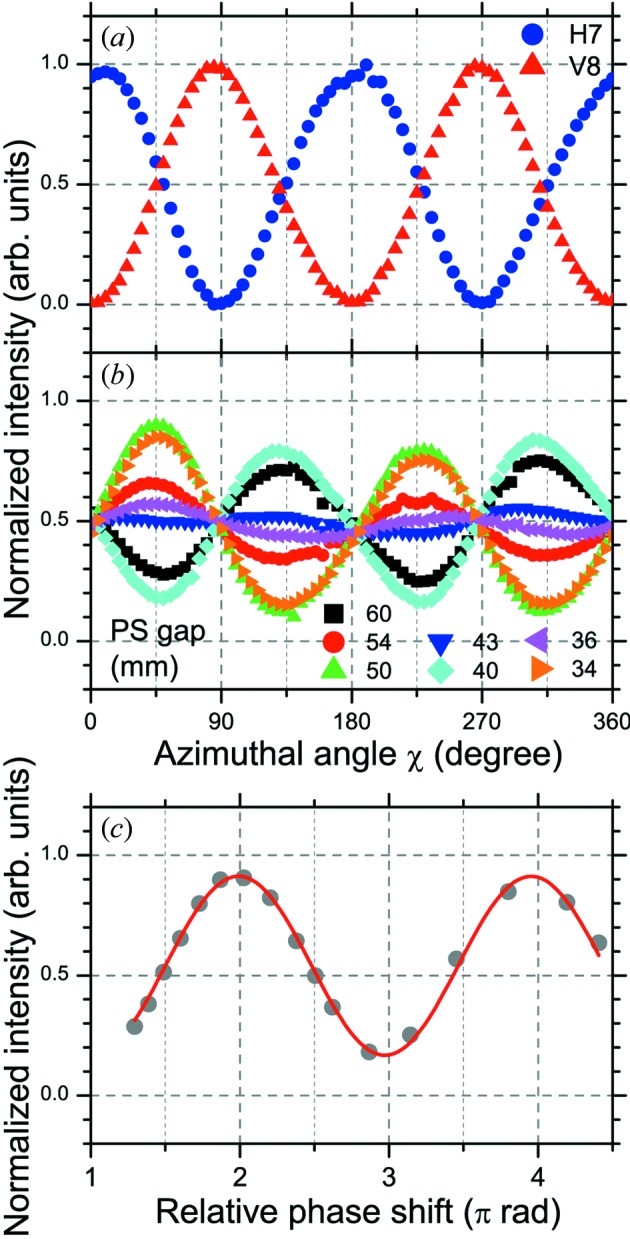
Normalized reflectivity curves of SR (*h*ν = 300 eV) emitted from (*a*) one horizontal (H7) or one vertical (V8) ID segment, (*b*) one horizontal and one vertical (H7 + V8) ID segments with different gap values of the phase shifter (PS7). (*c*) Intensities at analyzer azimuth angle of 45° in (*b*) are plotted as a function of relative phase shift in units of π rad. The relative phase shift is derived from the gap value of the PS. The curve is fitted with a sine function. All the measurements were performed at a photon energy of 300 eV using a 600 lines mm^−1^ grating with front-end slit opening of 0.5 mm × 0.5 mm and exit slit width of 100 µm.

To determine the degree of circular polarization *P*_C_, the circularly polarized light is first converted into linearly polarized light using the phase retarder. The procedure for deriving *P*_C_ in this optical configuration was explained in detail in the previous publication (Hirono *et al.*, 2005 ▶). Therefore, the procedure is described briefly here. The normalized Stokes parameter of transmitted light through the phase retarder, *S*′, is expressed as
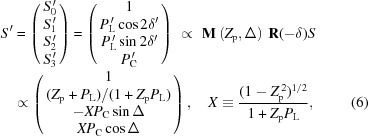
where **M** and **R** are Mueller matrices of the phase retarder and the analyzer, *Z*_p_ is the polarizance of the transmitting phase retarder, and Δ is the phase retardation angle introduced by the phase retarder. *P*_C_ is calculated from the 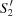 term in (6) and expressed as follows,
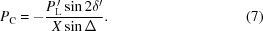



(ii) *Polarization analysis.* First, we show a typical example of polarization measurements using the rotating analyzer method. In Fig. 6(*a*) ▶, the reflected intensity of 300 eV SR emitted from one horizontal or one vertical ID segment is plotted as a function of azimuthal angle of the analyzer. The optical configuration in the measurements shown in Fig. 6 ▶ was that without the phase retarder, as shown in Fig. 5(*a*) ▶. The analyzer was the W/B_4_C multilayers (*N* = 100 and *d* = 2.99 nm) fixed at an incidence angle (measured from the surface normal) of 45°. Both reflectivity curves show a sinusoidal dependence on the azimuthal angle of the analyzer; as expected, they show a 90° phase difference between the horizontal and vertical linear polarizations. When one horizontal and one vertical ID segment are paired with one PM-PS placed between them, circularly polarized SR can be generated. Fig. 6(*b*) ▶ shows the reflectivity curves of 300 eV SR emitted from one horizontal and one vertical ID segment with different PM-PS gaps. The reflectivity curves in Fig. 6(*b*) ▶ are shifted by 45° with respect to those from the single horizontal or vertical ID segment in Fig. 6(*a*) ▶; the amplitude of the sinusoidal curves varies as a function of PM-PS gap. The PS introduces the relative phase shift of SR light emitted from each segment by changing the electron orbit between the ID segments. In Fig. 6(*c*) ▶, the intensities at an analyzer azimuth angle of 45° in Fig. 6(*b*) ▶ are plotted as a function of relative phase shift. The relative phase shift is calculated from the PM-PS gap value. The resulting curve can be fitted with a sine function. SR with phase shifts of (*n* + 1/2), (2*n*) and (2*n* + 1) [π rad] have circular, 45° linear and 135° linear polarization, respectively.

**Figure 7:**
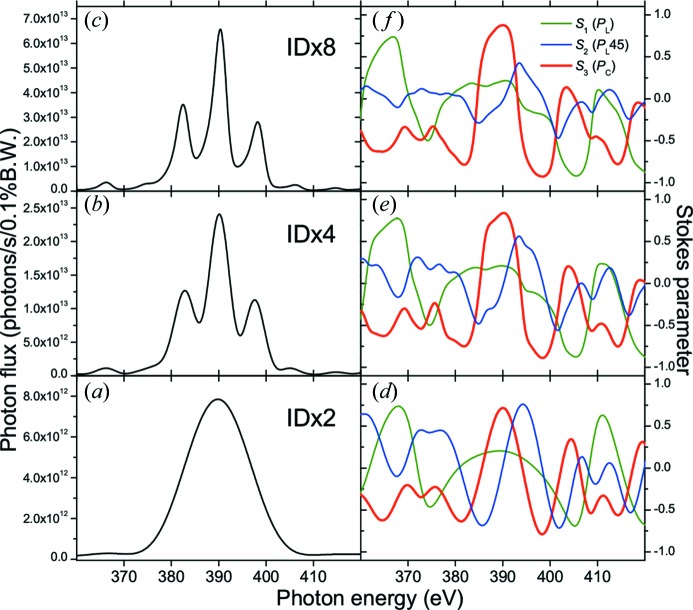
Simulated ID spectra and Stokes parameters for circularly polarized SR light (*h*ν ≈ 400 eV) emitted from two ID segments (ID × 2; *a*, *d*), four ID segments (ID × 4; *b*, *e*) and eight ID segments (ID × 8; *c*, *f*). The normalized Stokes parameters *S*_1_ (linear polarization; *P*_L_), *S*_2_ (linear polarization in a plane rotated by 45° with respect to *S*_1_ plane; *P*_L_45) and *S*_3_ (circular polarization; *P*_C_) are derived. In the simulation using *SPECTRA*, the distance between the light source point and the front-end slit was fixed at 44 m, and the opening size of the front-end slit was 0.5 mm × 0.5 mm; the ID segments were adjusted so that the side peak of the ID spectrum was located at 398 eV, and the phase shifters were set to produce the circular polarization. One should notice that the helicity of the circular polarization (*i.e.* the sign of *S*_3_) is opposite between the main peak and the side peak of the ID spectra.

Fig. 7 ▶ shows the ID spectra and Stokes parameters simulated by *SPECTRA* (Tanaka & Kitamura, 2001 ▶) for circularly polarized SR light (*h*ν ≈ 400 eV) emitted from two ID segments (*a*, *d*), four ID segments (*b*, *e*) and eight ID segments (*c*, *f*). The normalized Stokes parameters *S*_1_ (linear polarization; *P*_L_), *S*_2_ (linear polarization in a plane rotated by 45° with respect to the *S*_1_ plane; *P*_L_45) and *S*_3_ (circular polarization; *P*_C_) are derived. As the number of ID segments increases, the photon flux increases. Regarding the peak shape, the ID spectrum from two ID segments [Fig. 7(*a*) ▶] shows one broad peak, and the ID spectra from four or more ID segments [Figs. 7(*b*) and 7(*c*) ▶] show a characteristic peak shape consisting of a main peak and side peaks. In addition, the peak width of the ID spectra becomes narrower as the number of ID segments increases. The Stokes parameters for these different combinations of ID segments show that the degree of circular polarization (*S*_3_) for the side peak at higher energy in the ID spectrum is higher than that for the main peak. In addition, it should be noted that the helicity of the circular polarization (*i.e.* the sign of *S*_3_) is opposite between the main peak and the side peak of the ID spectra. Hereafter, the central peak with the maximum intensity is defined as the main peak, and the peak on the higher photon energy side is defined as the side peak.

**Figure 8:**
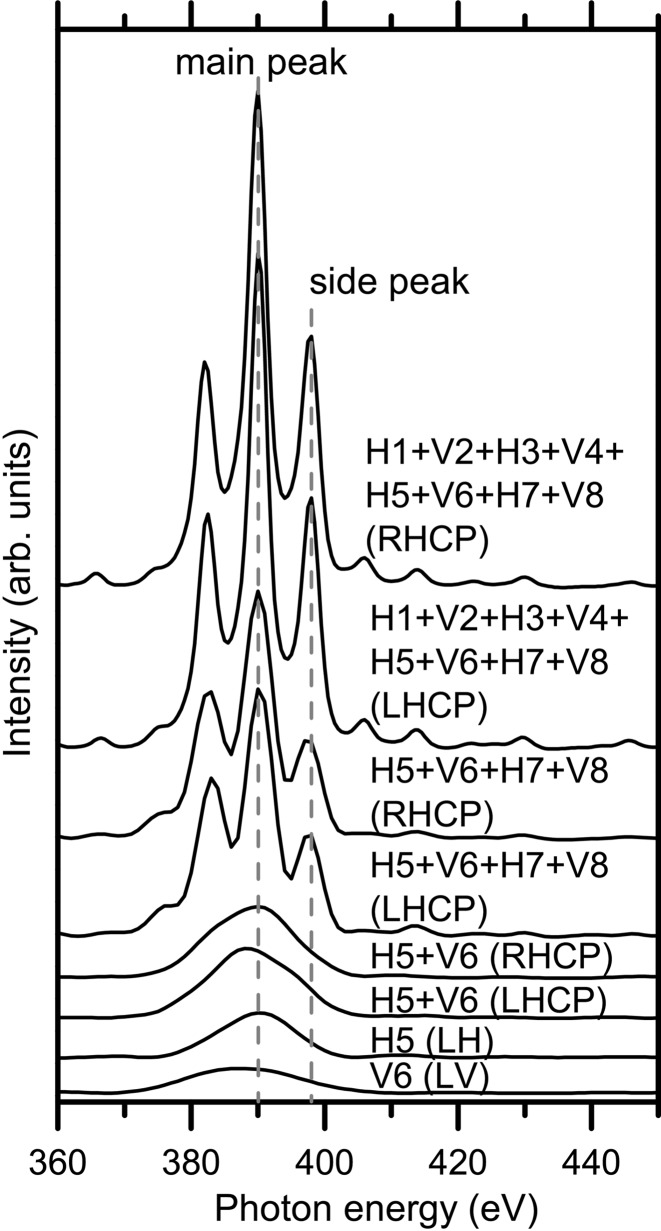
Experimentally obtained ID spectra with different combinations of ID segments at *h*ν ≈ 400 eV. All the spectra were measured with a 600 lines mm^−1^ grating, front-end slit opening of 0.5 mm × 0.5 mm and exit slit width of 100 µm. The following abbreviations are used in the figure: LH (linear horizontal), LV (linear vertical), RHCP (right-handed circular polarization), LHCP (left-handed circular polarization).

Fig. 8 ▶ shows the experimentally measured ID spectra (*h*ν ≈ 400 eV) with different combinations of ID segments. One ID segment having either horizontal (H5) or vertical (V6) polarization shows a broad ID spectrum; the horizontal ID segment (H5) yields a higher intensity than the vertical ID segment (V6). This is because the period of the horizontal ID segment (*N* = 26) is larger than that of the vertical ID segment (*N* = 20), as explained in Table 1 ▶. By combining horizontal and vertical ID segments, either left- or right-handed circularly polarized SR light can be generated. The ID spectrum from one horizontal and one vertical ID segment (H5 + V6) shows one broad peak. When the number of ID segments increases to four (H5 + V6 + H7 + V8) and eight (H1 + V2 + H3 + V4 + H5 + V6 + H7 + V8), the ID spectrum consists of a main peak and side peaks. The peak width of the ID spectra becomes narrower as the number of ID segments increases. These are consistent with the results of the *SPECTRA* simulation as shown in Fig. 7 ▶. Note that the ID spectra of circularly polarized SR light do not change when the helicity is changed between left-handed and right-handed.

**Figure 9:**
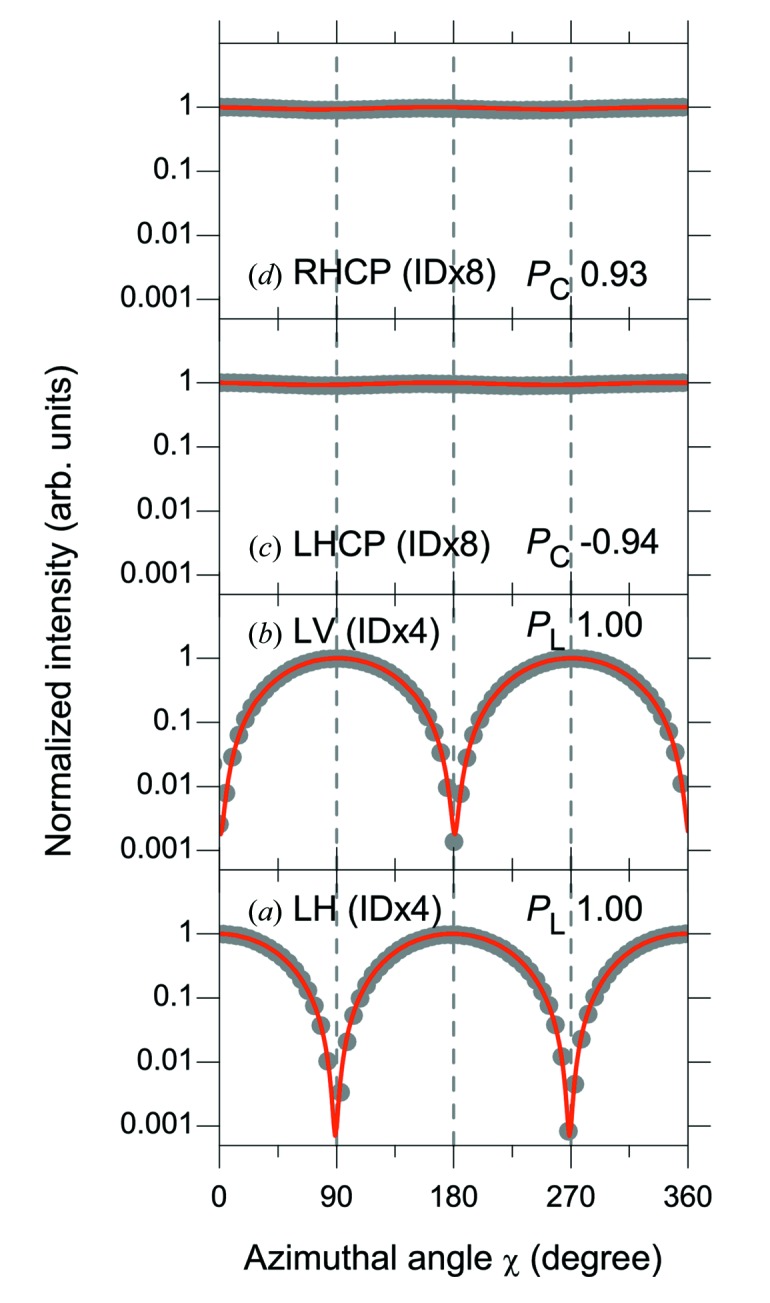
Normalized reflectivity curves of SR (*h*ν ≈ 400 eV) emitted from (*a*) four horizontal (H1 + H3 + H5 + H7) ID segments, (*b*) four vertical (V2 + V4 + V6 + V8) ID segments and (*c*, *d*) four horizontal and four vertical (H1 + V2 + H3 + V4 + H5 + V6 + H7 + V8) ID segments. For linearly polarized light in (*a*) and (*b*), the ID segments were adjusted so that the main peak of the ID spectrum was located at 398 eV; phase shifters were set to give the maximum photon flux. For circularly polarized light in (*c*) and (*d*), the ID segments were adjusted so that the side peak of the ID spectrum was located at 398 eV; phase shifters were set to produce the left- and right-handed circular polarization, respectively. The reflectivity curves were measured in the optical configuration without a phase retarder. The degree of linear polarization (*P*_L_) and the degree of circular polarization (*P*_C_) are derived and shown in the figure. All the measurements were performed at a photon energy of 398 eV using a 600 lines mm^−1^ grating with front-end slit opening of 0.5 mm × 0.5 mm and exit slit width of 100 µm. The following abbreviations are used in the figure: LH (linear horizontal), LV (linear vertical), RHCP (right-handed circular polarization), LHCP (left-handed circular polarization). The vertical axis is plotted on a logarithmic scale.

Fig. 9 ▶ shows the normalized reflectivity curves of SR (*h*ν ≈ 400 eV) emitted from (*a*) four horizontal (H1 + H3 + H5 + H7) ID segments, (*b*) four vertical (V2 + V4 + V6 + V8) ID segments, and (*c*, *d*) four horizontal and four vertical (H1 + V2 + H3 + V4 + H5 + V6 + H7 + V8) ID segments. For linearly polarized light in (*a*) and (*b*), the ID segments are adjusted so that the main peak of the ID spectrum is located at 398 eV; PSs are set to give the maximum photon flux. For circularly polarized light in (*c*) and (*d*), the ID segments are adjusted so that the side peak of the ID spectrum is located at 398 eV; PSs are set to produce left- or right-handed circular polarization. The reflectivity curves in Fig. 9 ▶ were measured without a phase retarder in the optical configuration of Fig. 5(*a*) ▶ to evaluate the degree of linear polarization. The reflectivity curves were also measured with a phase retarder in the optical configuration of Fig. 5(*b*) ▶ to evaluate the degree of circular polarization (not shown). The incidence angles (measured from the surface normal) of the analyzer (Sc/Cr, *N* = 200 and *d* = 2.24 nm) and the phase retarder (Sc/Cr, *N* = 300 and *d* = 3.15 nm) were fixed at 44.6° and 59.0°, respectively.

The normalized reflectivity curve was fit with equation (1) to obtain the contrast factor *C*, which was then divided by the experimentally determined polarizance *Z* of the analyzer (0.997) to derive the degree of linear polarization (*P*_L_). The degree of circular polarization (*P*_C_) was then obtained by the procedures described in §2.3.2(i). The degrees of linear polarization (*P*_L_) from four horizontal or four vertical ID segments in Figs. 9(*a*) and 9(*b*) ▶ are both 1.00. The degree of circular polarization (*P*_C_) from four horizontal and four vertical ID segments in Figs. 9(*c*) and 9(*d*) ▶ are −0.94 and 0.93 for left- and right-handed circularly polarized light, respectively.

Table 2 ▶ summarizes the photon flux intensity and degree of polarization for various SR light available at SPring-8 BL07LSU. The experimental and simulated values of the photon flux intensity and degree of polarization are compared at a photon energy of ∼400 eV. In addition, the characteristics of the main peak and side peak of the ID spectra with circular polarizations are compared.

First, let us examine the photon flux intensity. In the circular polarization, almost the same photon flux intensity is obtained in left-handed circular polarization (LHCP) and right-handed circular polarization (RHCP); for example, see the experimental photon flux intensity for LHCP (0.49) and RHCP (0.49) (ID × 8) at the side peak of the ID spectra. The photon flux intensity from the vertical ID segment is about half of that for the horizontal ID segment: LH (ID × 1) 0.10 and LV (ID × 1) 0.05. This is explained by the difference in the number of periods of the ID segments (*N* = 26 for horizontal and *N* = 20 for vertical). The photon flux density for a single ID segment is proportional to *N* ^2^: LH : LV = 26^2^ : 20^2^ = 1 : 0.592. The different angular dispersions of horizontal and vertical ID segments may also contribute to the ratio of the photon flux intensity between horizontal and vertical ID segments. As already seen for linear polarization in Fig. 4 ▶, the photon flux intensity of circularly polarized SR also increases in proportion to a value slightly smaller than *N* ^2^ when the number of ID segments increases: LHCP (ID × 8) : LHCP (ID × 4) : LHCP (ID × 2) = 1.01 : 0.53 : 0.15 = 6.73 : 3.53 : 1 at the main peak of the experimental ID spectra.

Next, we turn to the degree of polarization. The degree of polarization in the experiment is in good agreement with that in the simulation. In the circular polarizations, as expected from the simulation, the degree of circular polarization is higher for the side peak than for the main peak; for example, in RHCP (ID × 8), for the side peak, *P*_C_ = 0.93, and for the main peak, *P*_C_ = 0.88. The analysis of circular polarization is limited to the photon energies at which the SR phase retarder is available. In this study, circular polarization was analyzed only at ∼400 eV, which corresponds to the *L*-edge absorption of Sc in the Sc/Cr multilayer. In the linear polarizations, the degree of linear polarization (*P*_L_) is confirmed to be as high as 1.00 for both LH (ID × 4) and LV (ID × 4).

Therefore, we have confirmed the successful generation of soft X-rays with linear polarizations (horizontal and vertical) and circular polarizations (left-handed and right-handed) using the novel SCU. The degree of polarization evaluated experimentally using the polarimeter is in good agreement with that in the simulation.

## 3. Beamline monochromator   

In this section, we describe the design and performance (photon flux and energy resolution) of the beamline monochromator. The design will be explained briefly because it has been reported in our previous publication (Senba *et al.*, 2011 ▶).

### 3.1. Design   

**Figure 10:**
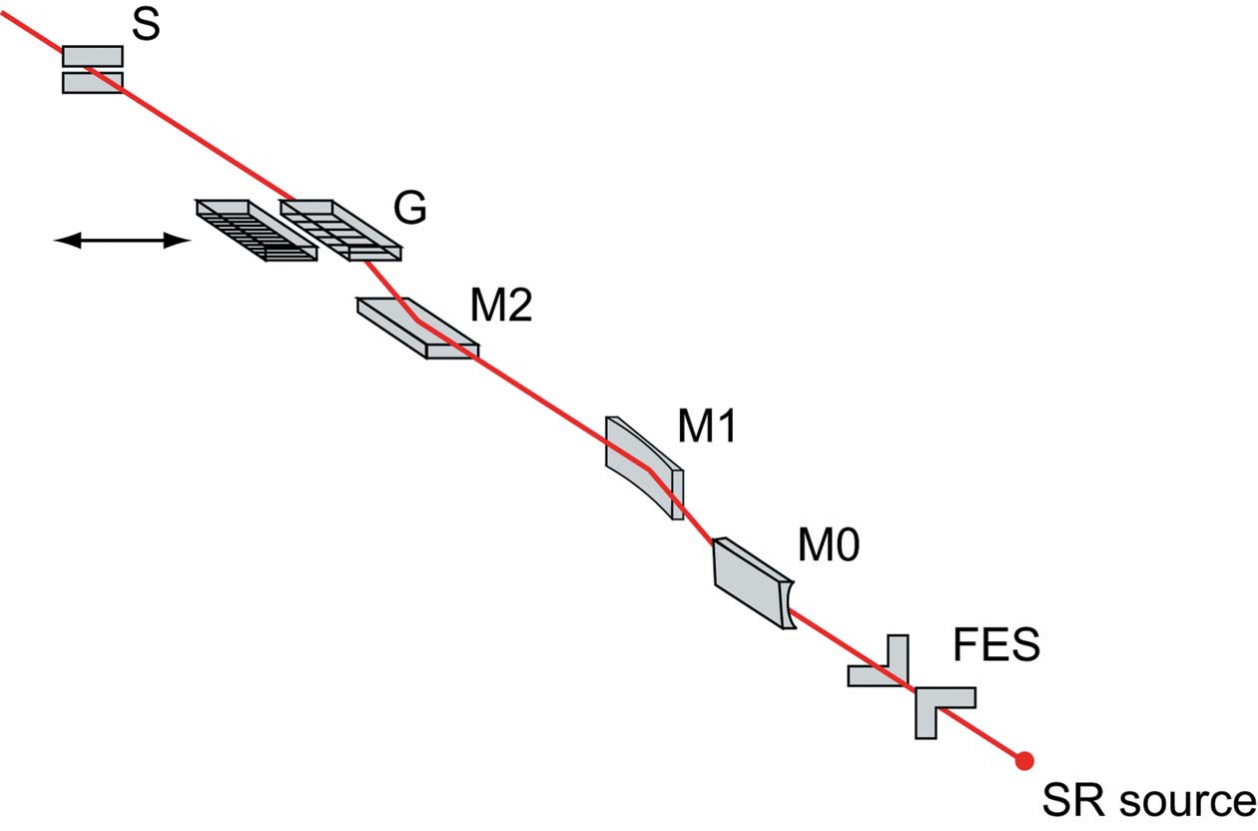
Optical layout of the beamline. Synchrotron radiation (SR) generated at an undulator passes through a front-end slit (FES), pre-focusing mirrors (M0 and M1), monochromator consisting of a mirror (M2) and gratings (G), and exit slit (S). Two varied-line-spacing gratings with center groove densities of 600 (G600) and 1200 (G1200) lines mm^−1^ are installed in the monochromator.

A variable-included-angle Monk–Gillieson mounting monochromator with a varied-line-spacing (VLS) plane grating (Amemiya & Ohta, 2004 ▶) has been adopted as a beamline monochromator. The optical layout of the beamline is schematically illustrated in Fig. 10 ▶; the optical parameters of the mirrors and gratings are summarized in Table 3 ▶.

The front-end slit (FES) is located at about 44 m from the center position of the SCU. It reduces the heat load on the beamline optics by cutting off the off-axis part of the undulator radiation (Oura *et al.*, 1998 ▶). The cylindrical mirror (M0) reflects the photon beam horizontally and focuses the beam vertically at the virtual focal point in front of the exit slit (S). The effects of both the surface slope error and the thermal deformation on the energy-resolving power are reduced by sagittal focusing. The bent-cylindrical mirror (M1) reflects the photon beam horizontally and focuses the beam horizontally on the exit slit. An included angle of 177.6° for M0 and M1 is selected considering the balance between the reflectivity and the heat load for all the mirrors and gratings, which are equipped with an indirect-water-cooled system. To reduce the heat load on the plane mirror (M2) and gratings (G), half of the reflecting surface of M1 is an Au-coated Si substrate, and the remaining half is uncoated Si. The Si surface is selected for use in the energy range *h*ν = 250–1500 eV to reduce the reflectivity of unnecessary higher-order photons. The plane mirror M2 varies the included angle of the grating in the range 172°–178° by off-axis rotation to optimize the resolving power. Note that the monochromator has no entrance slit.

Two VLS gratings with center groove densities of 600 (G600; Shimadzu) and 1200 (G1200; Horiba-Jobin-Yvon) lines mm^−1^ are currently installed in the monochromator. The local groove density of the VLS grating is expressed as *N*(*w*) = *N*_0_(1 + *a*_1_ *w* + *a*_2_ *w* ^2^ + *a*_3_ *w* ^3^ + …), where *a*_*i*_ are the groove density coefficients and *w* is the tangential distance from the center. *N*_0_ is thus the groove density at the center position (*w* = 0) of the grating (*i.e.* *N*_0_ = 600 for the 600 lines mm^−1^ grating). The included angle of the grating and the groove parameters were optimized by minimizing the aberrations according to a procedure described in the literature (Amemiya & Ohta, 2004 ▶), considering the reflectivity, diffraction efficiency and heat load. The entrance arm length *r*_1_ (the distance between the virtual focal point of M1 and the grating), the exit arm length *r*_2_ (the distance between the grating and the exit slit) and the groove density coefficients *a*_*i*_ were optimized for the two gratings. The optimized parameters for the two gratings are *r*_1_ = −15.34 m, *r*_2_ = 16 m, *a*_1_ = 1.2426 × 10^−4^ mm^−1^, *a*_2_ = 1.157 × 10^−8^ mm^−2^ and *a*_3_ = 9.559 × 10^−13^ mm^−3^. The surfaces of the two gratings are coated with gold for high reflectivity in the soft X-ray range. In addition, these two gratings can be interchanged by a motorized linear translational stage. Both the 600 and 1200 lines mm^−1^ gratings cover the entire energy range of *h*ν = 250–2000 eV.

### 3.2. Performance   

#### 3.2.1. Photon flux   

**Figure 11:**
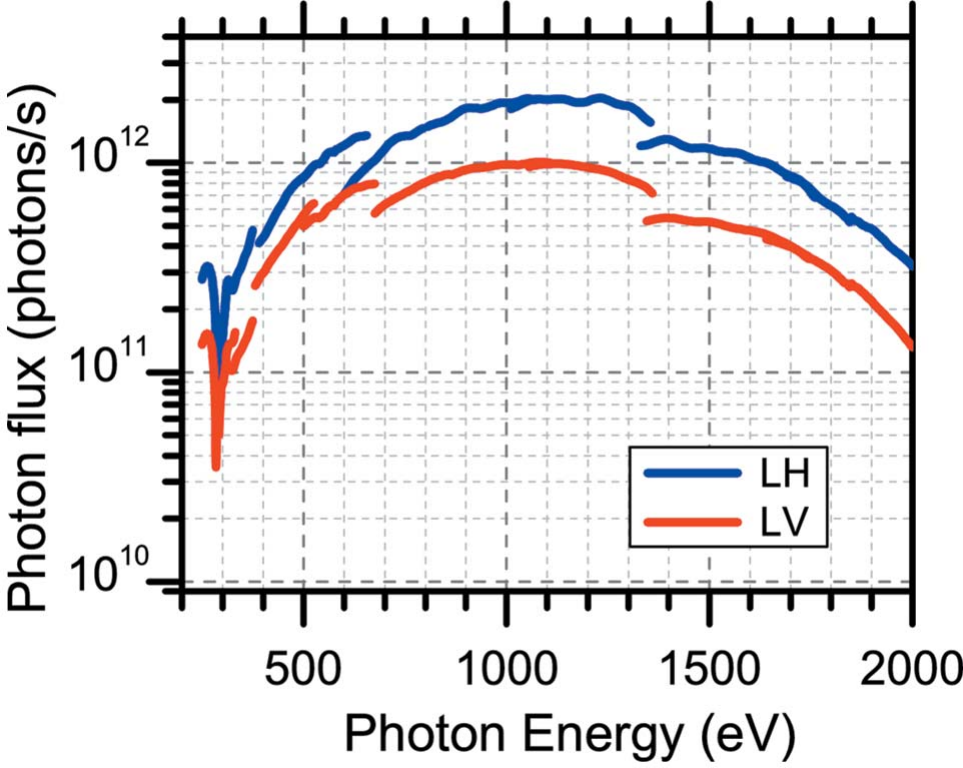
Experimentally obtained photon flux curves. Photon flux curves were measured with ID gap and grating scanned at fixed included angles (*i.e.* M2 mirror was fixed). Photon flux curves for linearly horizontally (LH) and linearly vertically (LV) polarized light are included. Note that four horizontal or four vertical ID segments are enabled for LH and LV polarizations, respectively. The grating was 600 lines mm^−1^ (G600). The opening of the front-end slit was 2.0 mm × 1.75 mm (height × width). The opening of the exit slit was 20 µm. Energy resolution measurements confirmed that the energy resolving power at photon energies of 250, 400, 540 and 640 eV exceeded 10000. The dip in the photon flux around 285 eV is due to carbon contamination of the optics (mirrors and grating).

Fig. 11 ▶ shows the photon flux curves measured at SPring-8 BL07LSU for both horizontally and vertically polarized light. The photon flux was measured with a photodiode (International Radiation Detectors, AXUV-100). The grating was 600 lines mm^−1^ (G600). The opening of the front-end slit was 2.0 mm × 1.75 mm (height × width). The opening of the exit slit was 20 µm. In the photon flux measurement, the photon energy was varied with the ID gap and the grating scanned at fixed included angles (*i.e.* M2 mirror was fixed). Although the energy resolving power changes when the photon energy is varied at a fixed included angle, the energy resolution measurements described in §3.2.2 confirmed that the energy resolving power at photon energies of 250, 400, 540 and 640 eV exceeded 10000. The dip in the photon flux around 285 eV originates from carbon contamination of the beamline optics (mirrors and grating). Removal of the contamination is planned by methods such as atmospheric pressure UV/ozone cleaning (Harada *et al.*, 1991 ▶) and *in situ* SR-activated oxygen cleaning (Toyoshima *et al.*, 2012 ▶).

#### 3.2.2. Energy resolution   

**Figure 12:**
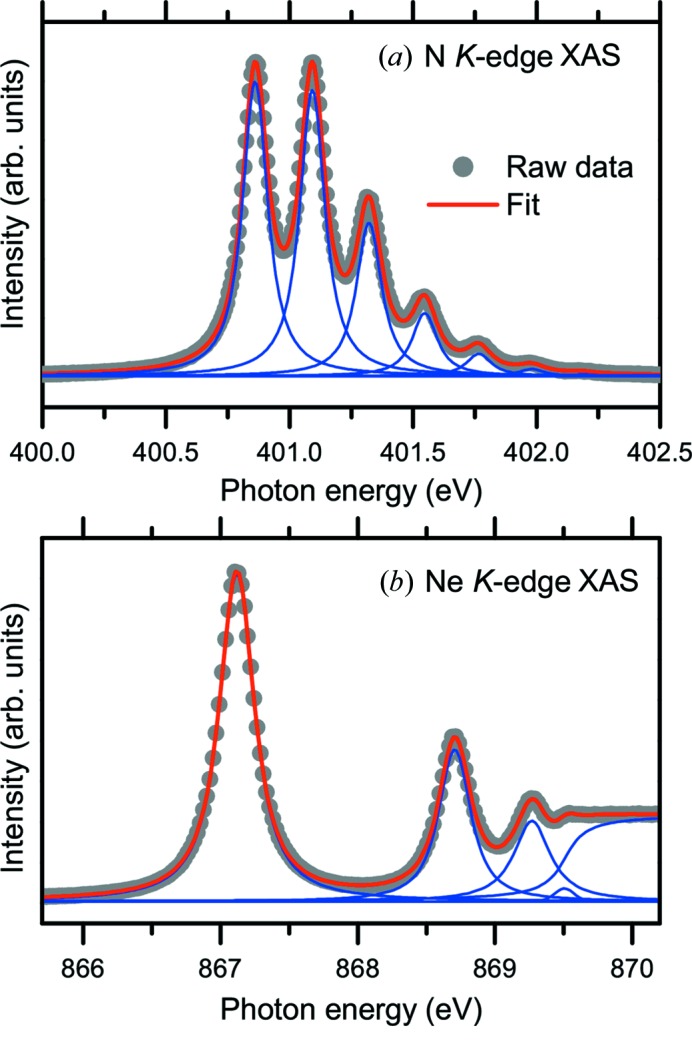
Total-ion-yield X-ray absorption spectra of (*a*) N_2_ gas at the N *K*-edge (*E*_*h*ν_ ≈ 401 eV) and (*b*) Ne gas at the Ne *K*-edge (*E*_*h*ν_ ≈ 867 eV). The grating used to measure the spectra was 600 lines mm^−1^ (G600). The opening of the exit slit was 10 µm. The energy resolving power (*E*_*h*ν_/Δ*E*) was estimated to be ∼8700 at the N *K*-edge and ∼10500 at the Ne *K*-edge from the FWHM of the Gaussian in the peak fitting using Voigt functions: (*a*) N_2_ *K*-edge, FWHM of Gaussian and Lorentzian were 38 and 114 meV; (*b*) Ne *K*-edge, FWHM of Gaussian and Lorentzian were 100 and 240 meV, respectively. The solid circles and solid line represent the raw experimental data and the result of peak fitting, respectively.

The energy resolution of soft X-rays at BL07LSU was evaluated and optimized using X-ray absorption spectroscopy (XAS) and X-ray photoemission spectroscopy (PES) spectra of gas molecules. Fig. 12 ▶ shows total-ion-yield XAS spectra of (*a*) N_2_ gas at the N *K*-edge (*E*_*h*ν_ ≈ 401 eV) and (*b*) Ne gas at the Ne *K*-edge (*E*_*h*ν_ ≈ 867 eV). The grating used to measure the XAS spectra was 600 lines mm^−1^. The opening of the exit slit was 10 µm. The energy resolving power (*E*_*h*ν_/Δ*E*) was estimated from the full width at half-maximum (FWHM) of the Gaussian in the peak fitting using a Voigt function. The N_2_ *K*-edge XAS spectrum in Fig. 12(*a*) ▶ is fitted with the Gaussian and the Lorentzian having FWHMs of 38 and 114 meV, respectively. The energy resolving power (*E*_*h*ν_/Δ*E*) is thus estimated to be ∼10500. The Ne *K*-edge XAS spectrum in Fig. 12(*b*) ▶ is fitted with the Gaussian and the Lorentzian having FWHMs of 100 and 240 meV, respectively. The energy resolving power (*E*_*h*ν_/Δ*E*) is thus estimated to be ∼8700. The energy resolution can be evaluated by XAS only at certain photon energies where the absorption edges of gas molecules are available. In addition, it is difficult to evaluate energy resolving powers greater than 10000 accurately using XAS spectra of gas molecules because the Lorentzian width of the lifetime broadening is larger than the Gaussian width of the photon resolution in these XAS measurements.

**Figure 13:**
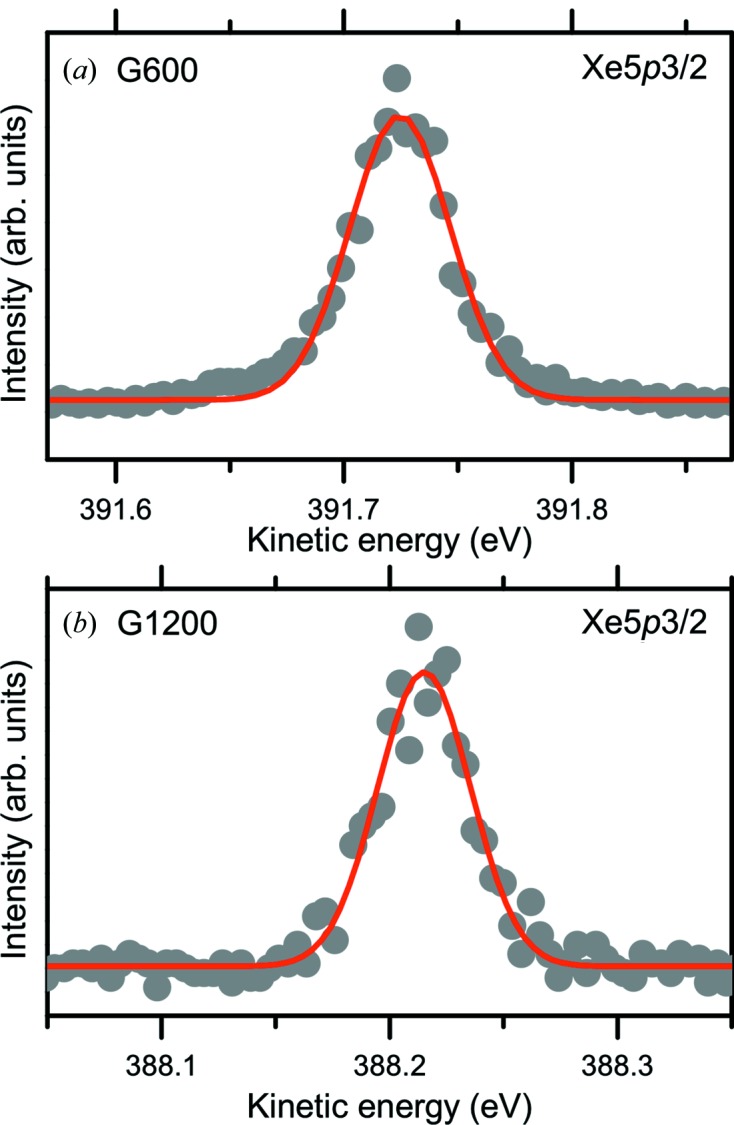
Xe 5*p* ^3/2^ photoemission spectra measured with gratings of (*a*) 600 lines mm^−1^ (G600) and (*b*) 1200 lines mm^−1^ (G1200) at a photon energy of 400 eV. The opening of the exit slit was 20 µm. The energy resolution (Δ*E*) and absolute energy (*E*_*h*ν_) of the X-rays were derived from the width and position of the Xe 5*p* ^3/2^ photoemission peak. The solid circles and solid line represent the raw experimental data and the result of peak fitting, respectively. Both the G600 and G1200 gratings achieved an energy resolving power (*E*/Δ*E*) of more than 10000, *i.e.* 12500 for G600 and 13600 for G1200.

To overcome these challenges, the PES spectra of Xe gas were measured to evaluate the energy resolution of soft X-rays using a high-energy-resolution electron analyzer (Ohashi *et al.*, 2001 ▶; Tamenori *et al.*, 2002 ▶). Fig. 13 ▶ shows the Xe 5*p* ^3/2^ PES spectra measured by a hemispherical electron analyzer (VG SCIENTA, SES-2002) with gratings of (*a*) 600 lines mm^−1^ (G600) and (*b*) 1200 lines mm^−1^ (G1200) at a photon energy of 400 eV. The opening of the exit slit was 20 µm.

The total linewidth (Γ_total_) of the PES spectrum of gas molecules is generally regarded as the convolution of the Gaussian and the Lorentzian. The Gaussian term consists of the photon resolution (Γ_*h*ν_), the resolution of the analyzer (Γ_analyzer_) and the Doppler broadening (Γ_D_). The Lorentzian term originates from the lifetime broadening (Γ_τ_). In Xe 5*p* PES spectra, the Lorentzian lifetime broadening is negligible because Xe 5*p* is the outermost valence shell of the Xe atom, so radiative and non-radiative decays of the ionic state are energetically prohibited, leading to the long lifetime of the excited ionic state. Therefore, only the Gaussian term contributes to the total linewidth of Xe 5*p* ^3/2^ PES spectra,
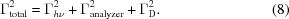
The photon resolution is thus derived as follows,
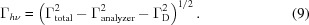
The Doppler broadening Γ_D_ due to the thermal motion of gas phase molecules is calculated as follows,
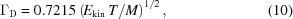
where Γ_D_ is in meV, *E*_kin_ is the kinetic energy of photoelectrons in eV, *T* is the gas temperature in K, and *M* is the molecular mass in atomic mass units (Baltzer *et al.*, 1993 ▶). Xe gas was chosen for use in the PES measurements because the Doppler broadening is small owing to its heavy mass; for example, the calculated Doppler broadening at *T* = 300 K and *E*_kin_ = 388 eV is 21.5 meV for Xe (*M* = 131.293) and 56.6 meV for Ne (*M* = 20.1797). The resolution of the analyzer was found to be 30 meV.

The energy resolution (Δ*E*) and absolute energy (*E*_*h*ν_) of the incident X-rays are derived from the width and position of the Xe 5*p* ^3/2^ photoemission peak. Both the G600 and G1200 gratings achieved an energy resolving power (*E*_*h*ν_/Δ*E*) of more than 10000, *i.e.* 12500 for G600 and 13600 for G1200 at a photon energy of 400 eV.

## 4. Endstations   

In this section, we briefly describe four endstations currently installed at SPring-8 BL07LSU. The experimental endstations were developed to conduct advanced soft X-ray spectroscopy for materials science, focusing in particular on the three important keywords in today’s science and technology: ‘smaller’, ‘faster’ and ‘more complex’. Detailed descriptions of the endstations can be found in our previous publications (Horiba *et al.*, 2011 ▶; Harada *et al.*, 2012 ▶; Ogawa *et al.*, 2012 ▶).

### 4.1. Time-resolved soft X-ray spectroscopy station   

The time-resolved soft X-ray spectroscopy station (Ogawa *et al.*, 2012 ▶; Yamamoto & Matsuda, 2013 ▶) enables time-resolved soft X-ray PES by combining a femtosecond laser with high-brilliant SR soft X-rays. The SR is a pulsed light source with a temporal pulse width of ∼50 picoseconds (ps; 10^−12^ s). In the time-resolved PES measurements, the transient electronic structures of materials after optical excitation by femtosecond laser pump pulses can be monitored in real time by picosecond soft X-ray probe pulses. The keyword emphasized at this station is thus ‘faster’. A two-dimensional (2D) angle-resolved time-of-flight (TOF) electron spectrometer (VG SCIENTA, ARTOF10k) is adopted as a high-transmission electron analyzer to solve the problem of the much lower count rate obtained in time-resolved experiments with a pump–probe method than in static experiments. In addition, the combination of the ARTOF10k instrument with soft X-rays enables simultaneous two-dimensional band mapping in a wide momentum space without rotating either the sample or the analyzer. The two independent light sources, *i.e.* femtosecond laser pump pulses and picosecond soft X-ray probe pulses, are synchronized, and their relative delay time is varied by a timing control unit. The timing jitter between the pump and probe pulses is much smaller than the pulse width of soft X-ray probe pulses (50 ps). This station is equipped with post-focusing mirrors so that the spot size of the soft X-ray beam is about 55 µm (horizontal) × 7 µm (vertical) at the sample position. The first application of the time-resolved PES system is a study of photo-excited carrier dynamics on semiconductor surfaces (Ogawa *et al.*, 2012 ▶, 2013 ▶). The carrier dynamics on semiconductors is highly relevant to photocatalysts and photovoltaics, in which energy is converted from photon energy to chemical or electrical energy through the generation of photo-excited carriers. Other applications of the time-resolved PES system include the study of photo-induced phase transitions and photo-induced chemical reactions.

### 4.2. Three-dimensional scanning photoelectron microscope station   

The three-dimensional scanning photoelectron microscope station (Horiba *et al.*, 2011 ▶) allows three-dimensional spatially resolved electron spectroscopy for chemical analysis (ESCA) of materials on a nanometer scale. We thus call this system 3D nano-ESCA. The keyword emphasized at this station is ‘smaller’. A soft X-ray beam is focused down to nanometer size on a sample using a Fresnel zone plate (FZP). The lateral (*x* and *y*) distribution of the electronic structures is obtained by scanning the sample using a two-axis piezo scanner and acquiring the photoelectron spectrum at each point. The depth (*z*) distribution of the electronic structures is obtained from the angular dependence of the photoelectron spectra using an angle-resolved photoelectron spectrometer (VG SCIENTA, R3000) with an extremely wide-angle lens (EWAL) and a two-dimensional (energy and angular distribution) detector. The acceptance angle of the EWAL is as large as 60°. The total spatial resolution of the system has been confirmed to be better than 70 nm. The targets of the 3D nano-ESCA system range from nanostructures such as semiconductor devices to nanoparticle catalysts and nanoscale phase separation phenomena. Further development towards measurements in a ‘more complex’ environment that allows *operando* analysis of device materials under an electrical bias is now underway.

### 4.3. Ultrahigh-resolution soft X-ray emission spectroscopy station   

The ultrahigh-resolution soft X-ray emission spectroscopy station (Harada *et al.*, 2012 ▶) features soft X-ray emission (SXE) spectroscopy with an ultrahigh energy resolving power (*E*/Δ*E*) of ∼10000 in the photon energy range 350–750 eV. SXE spectroscopy is a photon-in/photon-out bulk-sensitive technique that provides atomic and orbital specific information on the valence electronic structures of materials in various phases (*i.e.* solids, liquids and gases) (Kotani & Shin, 2001 ▶; Nilsson & Pettersson, 2004 ▶). The keyword of this station is ‘more complex’ environments. In addition, advances in the energy resolution of SXE spectroscopy allow the observation of low-energy (<0.5 eV) excitations such as electronic, vibrational, magnon, spinon and orbiton excitations (Ament *et al.*, 2011 ▶). The following three factors enable ultrahigh energy resolution: (i) an extremely small spot size (<1 µm) of soft X-rays focused by post-focusing mirrors in a Kirkpatrick–Baez configuration, (ii) the use of short entrance and long exit arms for the grazing-incidence flat-field-type optical mount spectrometer, which achieves high detection efficiency and high energy resolution, (iii) the application of the simplified coma-free operation mode proposed by Strocov *et al.* (2011 ▶). Note that the short entrance and long exit arms are advantageous because in our case the energy resolution is limited by the spatial resolution of the detector, not by the optical aberration (Harada *et al.*, 2012 ▶). The ultrahigh energy resolution of this station has attracted strong interest in studying the low-energy excitations in condensed matter such as a correlated electron system. The environmental versatility of SXE spectroscopy allows the study of electronic structures of liquids and solutions (Harada *et al.*, 2013 ▶), electrode materials in lithium ion batteries, and active sites for oxygen reduction in fuel cell catalysts under true atmospheric gas pressures and under electrochemical potentials (Niwa *et al.*, 2013 ▶).

### 4.4. Free-port station   

At the free-port station, users can bring their own experimental instrument and connect it to the beamline to perform experiments with high-brilliance SR soft X-rays. This station is equipped with post-focusing mirrors to realise a soft X-ray beam spot size of about 42 µm (horizontal) × 8 µm (vertical) at the sample position. Examples of experiments at the free-port station include 2D-PES with a wide acceptance angle of 90° and with image magnification capability using a new display-type ellipsoidal mesh analyzer (DELMA) (Goto *et al.*, 2011 ▶), and studies of X-ray magneto-optical effects using polarized soft X-rays.

## 5. Summary   

In summary, we have described the design and performance of the new soft X-ray beamline BL07LSU at SPring-8, which was constructed to perform advanced soft X-ray spectroscopy for materials science. The beamline is designed to achieve high energy resolution (*E*/Δ*E* > 10000) and high photon flux [>10^12^ photons s^−1^ (0.01% bandwidth)^−1^] in the photon energy range 250–2000 eV with controllable polarization. A new type of ID, the SCU, was developed and adopted as a light source at SPring-8 BL07LSU. The SCU consists of eight segments of horizontally/vertically polarized figure-8 undulators, seven PM-PSs, and seven EM-PSs. The successful operation of PM-PSs to both maximize the photon flux and control the polarization has been confirmed. According to XAS and PES spectra of gas molecules (Ne, N_2_ and Xe), the energy resolution was confirmed to exceed 10000. The photon flux was measured with a photodiode to achieve 10^12^ photons s^−1^ (0.01% bandwidth)^−1^ in almost all energy regions. The degree of linear polarization for the horizontal and vertical linearly polarized SR was evaluated using a soft X-ray polarimeter and found to be 1.00 with four horizontal ID segments and 1.00 with four vertical ID segments. The degree of circular polarization was measured using the polarimeter with a phase retarder to be −0.94 and 0.93 for left- and right-handed circularly polarized SR from four horizontal and four vertical ID segments, respectively.

## Figures and Tables

**Table 1:** Parameters of horizontal and vertical figure-8 undulators at SPring-8 BL07LSU Magnet period λ_u_ is defined as the period of the center-row magnet, which corresponds to the periodic length of the vertical (horizontal) magnet field in the case of the horizontal (vertical) figure-8 undulator. Note that electrons move along a single figure-8 orbit while they travel twice the period λ_u_.

	Horizontal figure-8	Vertical figure-8
Total length (m)	2.6	2.0
Magnet period λ_u_ (mm)	100	100
Number of periods	26	20
Minimum gap (mm)	28	20
Maximum gap (mm)	150	150
Deflection parameter		
*K*_*x*_	2.22	6.35
*K*_*y*_	6.59	2.74
Peak magnetic fields (T)		
*B*_*x*_	0.12	0.68
*B*_*y*_	0.71	0.15

**Table 2:** Summary of photon flux intensity and degree of circular or linear polarization (*P*_C_, *P*_L_) for various SR light available at SPring-8 BL07LSU Experimental (Exp) and simulated (Sim) values are compared at a photon energy of ∼400 eV. In the experiment, a 600 lines mm^−1^ grating was used with the front-end slit opening of 0.5 mm × 0.5 mm and exit slit width of 100 µm. In the simulation, the same front-end slit opening was adopted, and the distance between the light source point and the front-end slit was varied depending on the ID configuration. In both the experiment and the simulation, the ID segments and phase shifters were adjusted so that either the main peak or the side peak of the ID spectrum was located at 398 eV. The photon flux is normalized to that of RHCP (ID × 8) at the main peak of the ID spectrum. The following abbreviations are used: LH (linear horizontal), LV (linear vertical), RHCP (right-handed circular polarization), LHCP (left-handed circular polarization).

	ID configuration	Peak	Intensity (Exp)	Intensity (Sim)	*P*_C_, *P*_L_ (Exp)	*P*_C_, *P*_L_ (Sim)
LHCP (ID × 8)	H1 + V2 + H3 + V4 + H5 + V6 + H7 + V8	Side	0.49	0.40	−0.94	−0.92
RHCP (ID × 8)	H1 + V2 + H3 + V4 + H5 + V6 + H7 + V8	Side	0.49	0.42	0.93	0.93
LHCP (ID × 8)	H1 + V2 + H3 + V4 + H5 + V6 + H7 + V8	Main	1.01	1.02	−0.89	−0.87
RHCP (ID × 8)	H1 + V2 + H3 + V4 + H5 + V6 + H7 + V8	Main	1.00	1.00	0.88	0.88
LHCP (ID × 4)	H5 + V6 + H7 + V8	Side	0.25	0.22	−0.83	−0.88
RHCP (ID × 4)	H5 + V6 + H7 + V8	Side	0.25	0.23	0.85	0.88
LHCP (ID × 4)	H5 + V6 + H7 + V8	Main	0.53	0.51	−0.76	−0.83
RHCP (ID × 4)	H5 + V6 + H7 + V8	Main	0.53	0.51	0.75	0.83
LHCP (ID × 2)	H5 + V6	Side	0.07	0.06	−0.77	−0.78
RHCP (ID × 2)	H5 + V6	Side	0.07	0.07	0.79	0.78
LHCP (ID × 2)	H5 + V6	Main	0.15	0.14	−0.66	−0.71
RHCP (ID × 2)	H5 + V6	Main	0.15	0.14	0.69	0.71
LH (ID × 4)	H1 + H3 + H5 + H7	Main	0.73	0.71	1.00	1.00
LH (ID × 2)	H5 + H7	Main	0.32	0.24	1.00	1.00
LH (ID × 1)	H5	Main	0.10	0.09	0.99	1.00
LV (ID × 4)	V2 + V4 + V6 + V8	Main	0.40	0.51	1.00	1.00
LV (ID × 2)	V6 + V8	Main	0.18	0.17	0.98	0.99
LV (ID × 1)	V6	Main	0.05	0.06	0.95	0.98

**Table 3:** Parameters of optical elements at SPring-8 BL07LSU Shape, entrance arm length *r*_1_, exit arm length *r*_2_, incidence angle θ, and size of the optical elements in dimensions of length × width × height. VLS-PG: varied-line-spacing plane grating.

	Shape	*r*_1_ (m)	*r*_2_ (m)	θ (°)	Size (mm)	Coating
M0	Cylinder	49.50	23.34	88.80	400 × 50 × 50	Au
M1	Bent cylinder	52.50	21.00	88.80	400 × 50 × 30	Au and Si
M2	Plane	–	–	Variable	400 × 50 × 50	Au
Grating	VLS-PG	−15.34	16.00	Variable	150 × 50 × 50	Au
